# Evaluate the diagnostic and prognostic value of NUSAP1 in papillary thyroid carcinoma and identify the relationship with genes, proteins, and immune factors

**DOI:** 10.1186/s12957-022-02652-9

**Published:** 2022-06-16

**Authors:** Tiantian Gao, Lei Zhao, Fan Zhang, Conghui Cao, Shuting Fan, Xiaoguang Shi

**Affiliations:** 1 Department of Endocrinology, Shengjing Hospital of China Medical University, Shenyang, Liaoning Province 110001 People’s Republic of China; 2 Department of Laboratory Medicine, The First Affiliated Hospital of China Medical University, Shenyang, Liaoning Province 110001 People’s Republic of China; 3 Department of Endocrinology and Metabolism, Institute of Endocrinology, NHC Key Laboratory of Diagnosis and Treatment of Thyroid Diseases, The First Affiliated Hospital of China Medical University, Shenyang, Liaoning Province 110001 People’s Republic of China

**Keywords:** NUSAP1, Papillary thyroid carcinoma, Prognostic markers, Tumor immunity

## Abstract

**Background:**

Nucleolar spindle-associated protein 1 (NUSAP1) is reported to be a useful diagnostic and prognostic marker for a variety of cancers, but relevant studies are lacking in papillary thyroid carcinoma (PTC).

**Methods:**

The relationship between NUSAP1 expression and the overall survival (OS) of pan-cancer was examined by GEPIA and KMplot. We explored the relationship between NUSAP1 and clinical PTC data based on the THCA dataset of TCGA and the GEO dataset of NCBI; GO, KEGG analysis, and ceRNA networks were performed on co-expressed genes through LinkedOmics and Starbase. We assessed the relevance between NUSAP1 and the tumor microenvironment using ESTIMATE, correlations between NUSAP1 and immune cells with TIMER, the relationship between NUSAP1 and immunotherapy by TCIA, and small-molecule drugs targeting NUSAP1 that can be discovered using the CMap database.

**Results:**

Higher expression of NUSAP1 in pan-cancer tissues was correlated with shorter OS. NUSAP1 was also significantly expressed in PTC tissues and was an independent prognostic risk factor. Compared to the NUSAP1 low expression group, the NUSAP1 high expression group was more likely to also have lymph node metastasis, pathological PTC type, shorter progression-free survival (PFS), and higher scores for immune checkpoint inhibitor treatment. The genes associated with NUSAP1 were mostly involved in the cell cycle, immune-related pathways, and AITD. Ten lncRNAs (GAS5, SNHG7, UCA1, SNHG1, HCP5, DLEU2, HOTAIR, TP53TG1, SNHG12, C9orf106), eleven miRNAs (hsa-miR-10a-5p, hsa-miR-10b-5p, hsa-miR-18a-5p, hsa-miR-18b-5p, hsa-miR-128-3p, hsa-miR-214-3p, hsa-miR-219a-2-3p, hsa-miR-339-5p, hsa-miR-494-3p, hsa-miR-545-3p, hsa-miR-769-5p), and one mRNA (NUSAP1) were constructed. NUSAP1 participated in the formation of the tumor microenvironment. CMap predicted the 10 most important small molecules about NUSAP1.

**Conclusions:**

In PTC, NUSAP1 shows good diagnostic value and prognostic value; NUSAP1 impacts the cell cycle, immune-related pathways, and AITD and has a complex effect on the tumor microenvironment in PTC.

**Supplementary Information:**

The online version contains supplementary material available at 10.1186/s12957-022-02652-9.

## Introduction

Thyroid cancer is a common endocrine malignancy. According to the World Health Organization, papillary thyroid carcinoma (PTC) is the most common histological type of thyroid cancer [1]. The incidence of thyroid cancer has increased in recent years, with the most prominent changes occurring in the incidence of PTC [2]. Previous studies [3] have indicated that the recurrence rate of PTC reaches 28% and is related to age, tumor type, and disease stage. Relapse is more likely with increased age, follicular papillary thyroid carcinoma (FVPTC), T4 stage, and cervical lymph node involvement with distant metastasis. Therefore, it is necessary to study and identify high-risk patients in order to ensure appropriate treatment [4]. PTC has been shown to be more prevalent among people with autoimmune diseases. In a prospective cohort study of 9851 subjects, Morais et al. [5] demonstrated that the risk of malignant nodules increased significantly when the patient had autoimmune thyroiditis (AIT) as compared to those who did not have the disease. Briseis et al. [6] conducted a prospective study of about 50,000 patients with follow-up for 10 years. Compared with people without diabetes, those with diabetes had a significantly higher risk of developing thyroid cancer. Furthermore, diabetes has been linked to differentiated thyroid cancer. Although previous studies have demonstrated that autoimmune diseases increase the risk of PTC, there are few molecular mechanisms to explain this. Therefore, it is necessary to study the mechanism of PTC in-depth to better understand this condition.

Nucleolar spindle-associated protein 1 [7] (NUSAP1) is a protein-related to cell proliferation. This protein is selectively expressed during cell proliferation and reaches a peak at the transition from interphase to mitosis and is then degraded during cell division. It was first reported in 2007 that expression of NUSAP1 was increased in melanoma compared to normal tissue [8]. A number of recent studies have shown the involvement of NUSAP1 in cancer and have explored its mechanism of action in various diseases, including glioblastoma [9], bladder cancer [10], nasopharyngeal carcinoma [11], non-small-cell lung cancer [12], liver cancer [13], osteosarcoma [14], ovarian cancer [15], breast cancer [16], and gastric cancer [17]. The report by Guo et al. [18] demonstrated that NUSAP1 induces the activation of the Hippo signaling pathway by promoting the stability of Yes1-associated transcriptional regulator (YAP1) protein, thereby promoting the growth and invasion of gastric cancer cells and tumor growth. It has also been reported that silencing NUSAP1 reduces the Wnt/β-catenin signaling pathway in nasopharyngeal cancer by downregulating phosphorylation of glycogen synthase kinase 3 beta (GSK-3β), reducing cell proliferation, and reducing invasion [11]. In 2011, Salmena et al .[19] proposed that competing endogenous RNAs (ceRNAs) can regulate gene expression by binding to the same miRNA. A growing number of researchers demonstrated that ceRNAs were involved in the development of various tumors. However, few studies have been conducted on the role of the NUSAP1-related ceRNA regulatory network in PTC.

Immunotherapy has brought a huge revolution to the treatment of cancer. Recent developments in immune checkpoint blockade therapy (ICB) have resulted in increased success for patients with CD274 (also known as PDL1), PDCD1 (also known as PD1), and CTLA4 [20]. Mehnert et al. [21] found through a clinical trial on PTC (NCT02054806) that pembrolizumab (PD-1 inhibitor) not only exerts anti-tumor activity, but also has controllable safety. French [22] concluded that a large amount of data is currently accumulating on the use of single-agent ICB therapy and ICB combination therapy to treat thyroid cancer. Therefore, it is necessary to find some new small-molecule drugs.

Extensive research has been conducted on NUSAP1, but the research value of NUSAP1 in PTC remains unclear. Therefore, in the current study, we used bioinformatics to conduct functions and therapy studies related to NUSAP1 in PTC to lay a theoretical foundation for subsequent research.

## Materials and methods

### Dataset

We downloaded The Cancer Genome Atlas Thyroid Cancer (TCGA THCA) dataset, including miRNA expression, clinical data, and gene expression, from the University of California at Santa Cruz (UCSC) Xena browser^1^. The dataset included 59 normal thyroid tissues and 497 PTC tissues.

For evaluating the diagnostic value of NUSAP1, we downloaded data from the National Center for Biotechnology Information (NCBI) Gene Expression Omnibus (GEO) database^2^. The download included normal thyroid tissues and PTC tissues. The dataset included GSE27155 (N = 4, T = 51), GSE33630 (N = 45, T = 49), GSE58545 (N = 18, T = 27), and GSE60542 (N = 30, T = 33).

### The expression of NUSAP1 in pan-carcinoma

The Tumor Immune Estimation Resource (TIMER) website was used to analyze the expression of NUSAP1 in other cancers and its correlation with the abundance of six types of tumor immune cells. The Gene Expression Profiling Interactive Analysis (GEPIA)^3^ [23] includes RNAseq data and clinical data from TCGA and the Genotype-Tissue Expression project (GTEx), which can be used to analyze the relationship between NUSAP1 expression and overall survival (OS) in other cancers. The KMplot database was used to validate the prognosis of NUSAP1 in other cancers [24].

### The expression of NUSAP1 in different datasets of PTC

First, NUSAP1 mRNA levels were compared in normal thyroid tissue and PTC tissue through the THCA dataset. The diagnostic value of NUSAP1 was evaluated by receiver operating characteristic (ROC). The GSE27155, GSE33630, GSE58545, and GSE60542 datasets were used to further analyze the mRNA level and diagnostic value of NUSAP1. According to the upper quartile of NUSAP1 in the THCA dataset, samples were categorized into the L-NUSAP1 group or H-NUSAP1 group. Samples below the upper quartile were placed in the L-NUSAP1 group, and samples higher than the upper quartile were defined as the H-NUSAP1 group. Kaplan-Meier (K-M) survival curves were evaluated by the log-rank test. The endpoint of this study was progression-free survival (PFS). A heat map was used to visualize the differences in the clinical data between the L-NUSAP1 and H-NUSAP1 groups.

### Analysis of the underlying mechanism of NUSAP1

The LinkedOmics website^4^ is a multi-omics database that includes 32 types of cancer, with data from TCGA and the Clinical Proteomics Tumor Analysis Consortium (CPTAC) [25]. We used this website to analyze the molecules that were co-expressed with NUSAP1 and visualized expression using volcano maps and heat maps. According to the Pearson correlation coefficient, the first 1000 genes positively correlated with NUSAP1 and the first 1000 genes negatively correlated with NUSAP1 were selected; a PPI network was constructed using String^5^ (version 11.0; interaction value = 0.99). A Cytoscape was used to visualize (version 3.8.0) and display the core genes at the same time. Finally, GO analysis and KEGG analysis were performed by gene set enrichment analysis (GSEA).

### Analysis of NUSAP1 and tumor microenvironment

ESTIMATE [26] was based on the ssGSEA algorithm to score the matrix and immune gene sets. Immune cell infiltration (immuneScore), stromal content (stromalScore), and tumor purity were calculated for each THCA sample through the “estimate” R package. The TIMER^6^ website [27] is a tumor-infiltrating immune cell component analysis website for analyzing the correlation between the expression of NUSAP1 and the abundance of six tumor-infiltrating immune cells. The *p*-values and correlation values (cor) were obtained using Spearman’s rank correlation test after purity adjustment. Scatter plots were used to display NUSAP1 and immune cell biomarkers [B cell (CD19 and CD79A), CD8+ T cell (CD8A and CD8B), CD4+ T cell (CD4), M1 macrophage (NOS2, IRF5, and PTGS2), and M2 macrophage (CD163, VSIG4, and MS4A4A), neutrophil (CEACAM8, ITGAM, and CCR7), and dendritic cell (HLA-DPB1, HLA-DQB1, HLA-DRA, HLA-DPA1, CD1C, NRP1, and ITGAX)].

### Construction of the ceRNA network

StarBase v2. 0[28] searches for micorRNA targets through high-throughput CLIP-Seq experimental data and degradome experimental data was used to predict mRNAs. Intersecting genes predicted to bind to NUSAP1 and differentially expressed in PTC were considered as potential miRNAs and lncRNAs in PTC. Finally, a lncRNA-miRNA-mRNA network was constructed, and the data were visualized using the Cytoscape software 3.8.0.

### The value of NUSAP1 in PTC immunotherapy

The correlation between NUSAP1 and immune checkpoints (CD274, CTLA4, and PDCD1) was analyzed through the GEPIA website.

TCIA^7^ [29] contains medical images of many tumors along with clinical information. The difference between PD1 and CTLA4 treatments in the L-NUSAP1 group and the H-NUSAP1 group was analyzed and visualized through the TCIA website. The “RColorBrewer” R package was used to analyze the differences between the L-NUSAP1 and H-NUSAP1 groups in different immunotypes (C1, C2, C3, C4, C5, and C6).

### Small-molecule drugs identified by connectivity map

Genes related to NUSAP1 were identified by LinkedOmics. CMap [30] query was performed on inputting the top 150 positive and top 150 negatively related genes to find small molecules with the same effect as NUSAP1. Negatively related drugs that reverse the effect of NUSAP1 were found in the top 10.

### Statistical analysis

Differences in NUSAP1 expression in normal thyroid tissue and PTC tissue were analyzed using the Mann-Whitney test. Survival analysis was performed by the log-rank test. The difference in clinical information between the L-NUSAP1 and H-NUSAP1 groups was analyzed using the chi-squared test, and the correlation was analyzed using the Spearman correlation. Data were processed through R (v4.2.2) and SPSS version 25.0. Data were visualized with R (v4.2.2) and GraphPad Prism V.9.0 software.

## Results

### The expression of NUSAP1 and pan-cancer

**Fig. 1:**
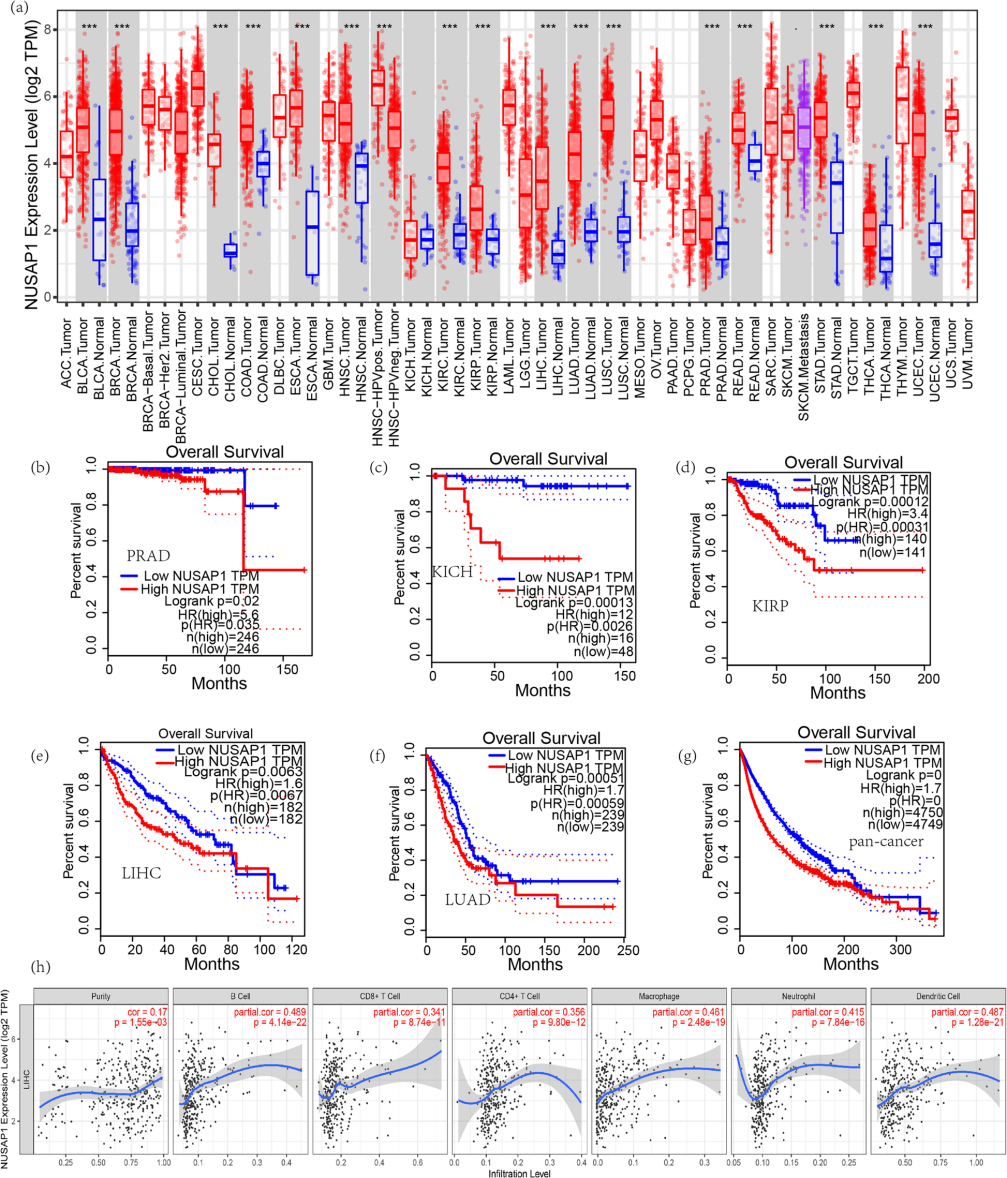
Analysis of NUSAP1 in pan-cancer. **a** TIMER was used to examine the expression of NUSAP1 in 32 cancers, as seen in a box diagram. Survival analysis in prostate adenocarcinoma (PRAD) (**b**), renal chromophobe cell carcinoma (KICH) (**c**), renal papillary cell carcinoma (KIRP) (**d**), hepatocellular carcinoma (LIHC) (**e**), lung adenocarcinoma (LUAD) (**f**), and pan-cancer (**g**) were shown. The horizontal axis represents the overall survival rate, and the vertical axis represents the month. **h** TIMER was used to analyze the correlation between the expression level of the NUSAP1 and the infiltration level of six immune cells in LIHC. *p < 0.5, **p < 0.01,  ***p < 0.001

In order to explore the expression of NUSAP1 in cancer, we analyzed its expression level, prognostic value, and relationship with the six main immune cells. First, we analyzed the expression of NUSAP1 in pan-cancers and visualized the expression of NUSAP1 in 32 cancers and 2 cancer subtypes through box plots. As shown in Fig. 1a, the expression level of NUSAP1 was increased significantly in 16 cancers, highlighted by a gray background color. Metastatic skin melanoma cancer was more common than skin melanoma cancer. But there was no significant change in 16 cancers, so only the expression of NUSAP1 in cancer tissues is shown. For high-expressing cancers, the expression in different subtypes is further analyzed. Among them, the HPV-positive group of head and neck squamous cell carcinoma is higher than the HPV-negative group, and the difference in the three subtypes of breast cancer is not very significant. The prognostic value of NUSAP1 in these 16 cancers was analyzed by log-rank test. The results showed that NUSAP1 was related to five cancers; prostate adenocarcinoma (PRAD), kidney chromophobe (KICH), kidney renal papillary cell carcinoma (KIRP), liver hepatocellular carcinoma (LIHC), and lung adenocarcinoma (LUAD) were associated with shorter OS (Fig. 1b–f). Out of 9499 cases of pan-cancer, cancer risks in the high-expressing NUSAP1 group were 1.7 times that of the low-expressing NUSAP1 group (*p* < 0.0001) (Fig. 1g). Immune cells in LIHC were most closely associated with NUSAP1, with B cells, DC cells, and macrophages being the most obvious (Fig. 1h). To further investigate the prognostic potential of NUSAP1 in cancer, we used the KMplot database to study NUSAP1 in cancer (Additional file 1: Fig. S1). We found that high NUSAP1 expression was also closely related to the poorer prognosis of OS in KIRC, KIRP, LIHC, and LUAD. Besides, pancreatic ductal adenocarcinoma (PDAC) had the same result.

### The expression and diagnostic value of NUSAP1 in PTC

**Fig. 2:**
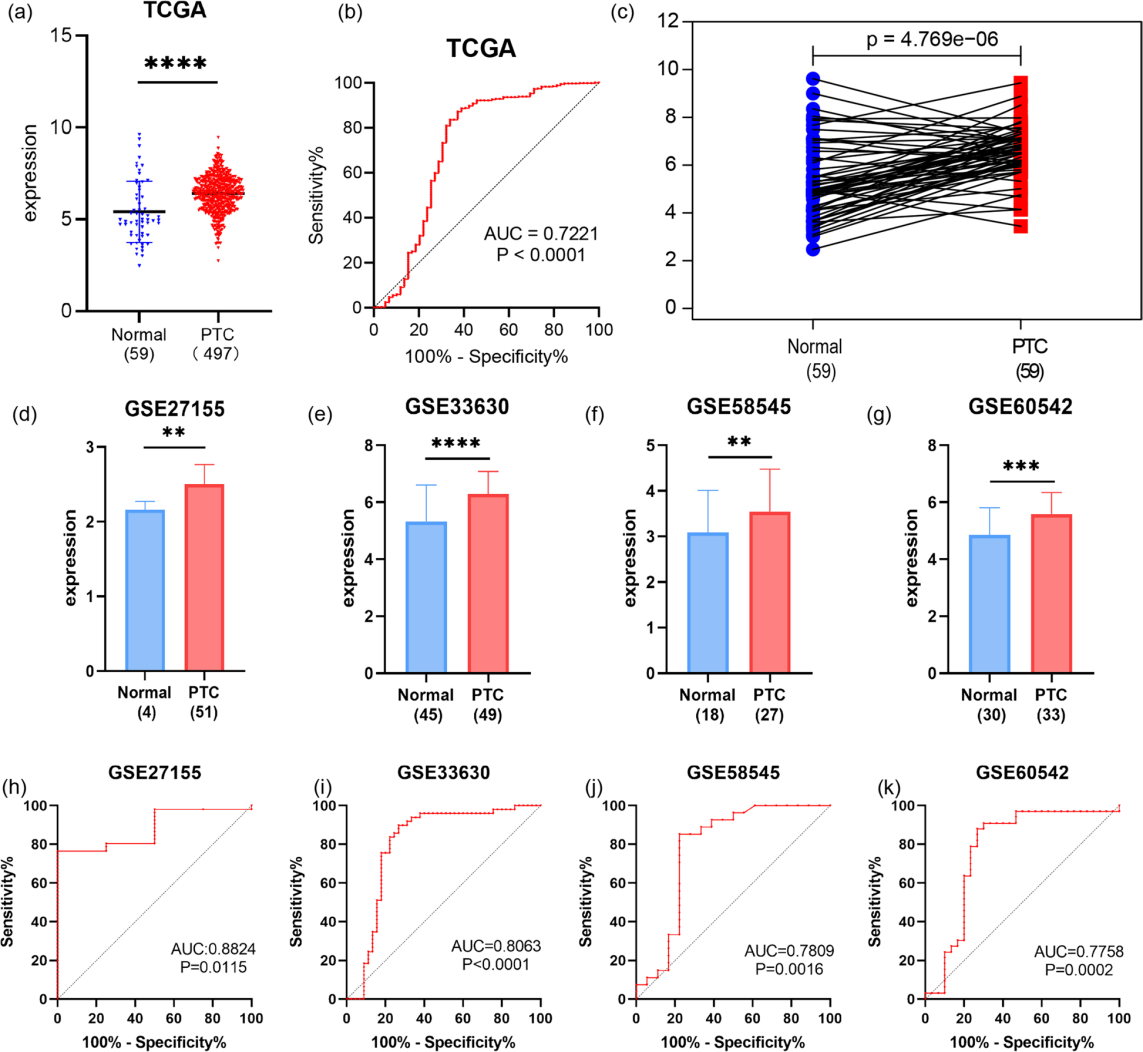
Expression and diagnostic value of NUSAP1 in PTC. **a** Scatter plot of NUSAP1 mRNA levels in normal thyroid tissue and PTC in TCGA. **b** ROC curve shows the diagnostic value of NUSAP1. **c** mRNA levels in matched normal thyroid and PTC tissues are analyzed and based on TCGA. **d**–**g** The GSE27155, GSE33630, GSE58545, and GSE60542 datasets verify the mRNA levels of NUSAP1 in PTC. **h**–**k** The ROC curve was used to verify the diagnostic value of NUSAP1 through the GEO dataset. *p < 0.05, **p < 0.01, ***p < 0.001, ****p < 0.0001

The THCA dataset was used to examine the NUSAP1 mRNA levels in normal thyroid tissue and PTC. As illustrated in Fig. 2a, the expression of NUSAP1 was higher in PTC compared with normal thyroid tissue (*p* < 0.0001). To determine the diagnostic value, we plotted the ROC curve and calculated its area as 0.7221 (*p* < 0.0001), indicating that it had a good diagnostic value (Fig. 2b). For the purpose of excluding the influence of individual differences, the THCA dataset was utilized to conduct a paired analysis. The results showed that NUSAP1 expression was increased in 59 paired PTC samples (Fig. 2c, *p* < 0.0001]. In order to verify the above results, we download the GSE27155, GSE33630, GSE58545, and GSE60542 datasets from the GEO database. As shown in Fig. 2d in the GSE27155 dataset, the expression of NUSAP1 in PTC was significantly higher than that in normal thyroid tissue (*p* < 0.0076), which was consistent with the results from other datasets [GSE33630 (*p* < 0.0001), GSE58545 (*p* <0.0012), GSE60542 (*p* < 0.0001)] (Fig. 2e–g). The areas under the ROC curve were 0.8824 (*p* < 0.0115), 0.8063 (*p* < 0.0001), 0.7809 (*p* < 0.0018), and 0.7758 (*p* < 0.0002); all findings support the above results (Fig. 2h–k).

### The prognostic value and clinical value of NUSAP1

**Fig. 3:**
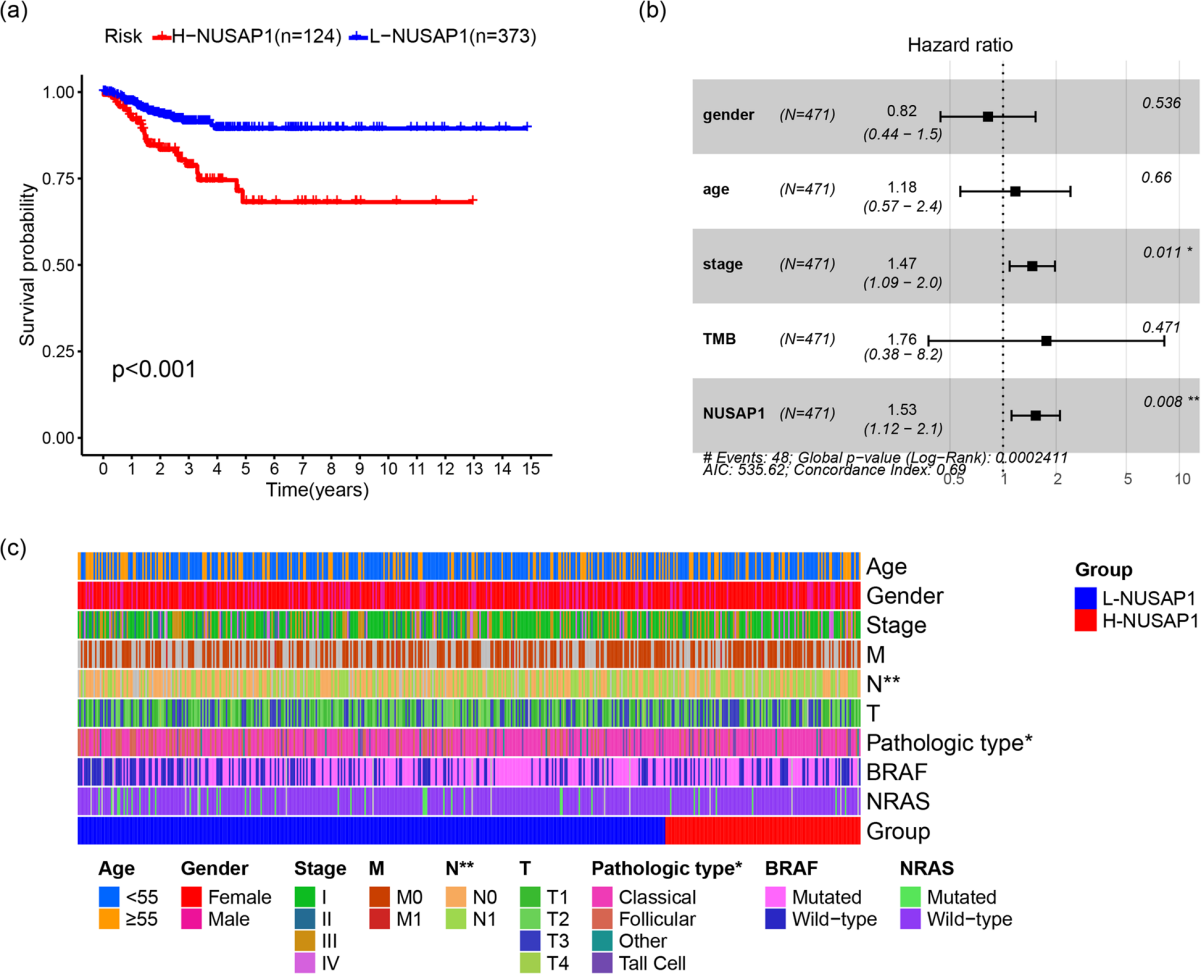
The prognostic value and clinical value of NUSAP1. **a** Survival analyses between the L-NUSAP1 (*n* = 373) and H-NUSAP1 (*n* = 124) groups in PTC with positive results are given. **b** Multivariate Cox regression results of NUSAP1 are shown by forest plot. **c** A heat map shows the clinical data of the L-NUSAP1 and H-NUSAP1 groups. The following figure displays the colors of different clinical data. *p < 0.05, **p < 0.01

In order to detect the prognostic value of NUSAP1 in PTC, the THCA dataset was got from the TCGA database and divided into L-NUSAP1 (*n* = 373) and H-NUSAP1 (*n* = 124), according to the NUSAP1 expression level. KM survival analysis showed that the progression-free survival (PFS) of the H-NUSAP1 group was shorter (*p* < 0.0001) compared with the L-NUSAP1 group (Fig. 3a). By regression analysis, we determined that age (*p* < 0.008), tumor stage (*p* < 0.001), tumor mutation burden (TMB, *p* < 0.004), and NUSAP1 expression (*p* < 0.005) were different from PFS (Table 1). Multivariate Cox regression analysis revealed that NUSAP1 may be an independent prognostic risk factor (HR = 1.5328, 95%CI 1.1171–2.1031, *p* = 0.0081) after adjusting for gender, age, stage, and TMB (Fig. 3b). In Cox regression, for dichotomous variables, we used males and < 55 years as the reference group, respectively. To determine the clinical value of NUSAP1 in PTC, the chi-squared test was applied to explore the diversity in various clinical data between the L-NUSAP1 and H-NUSAP1 groups (Table 2). The results showed that the H-NUSAP1 group was more prone to lymph node metastasis (*p* < 0.013) and more typical PTC types (*p* < 0.05) (Fig. 3c).

**Table 1:** Univariate and multivariate Cox regression results

AAW	Univariate analysis		Multivariate analysis	
	Hazard ratio (95%CI)	*p*-value	Hazard ratio (95%CI)	*p*-value
Gender	0.681 (0.374–1.242)	0.210	0.822 (0.442–1.529)	0.536
Age	2.166 (1.228–3.821)	0.008	1.175 (0.572–2.412)	0.660
Stage	1.598 (1.255–2.035)	0.000	1.466 (1.090–1.970)	0.011
TMB	5.863 (1.765–19.472)	0.004	1.762 (0.378–8.217)	0.471
NUSAP1	1.550 (1.139–2.109)	0.005	1.533 (1.117–2.103)	0.008

**Table 2:** Relationship between NUSAP1 and clinical data

Clinical parameters	L-NUSAP1 (*n* = 373, %)	H-NUSAP1 (*n* = 124, %)	*p*-value
Age (years)			
< 55	249 (66.8)	83 (66.9)	1
≥ 55	124 (33.2)	41 (33.1)	
Gender			
Female	271 (72.7)	92 (74.2)	0.816
Male	102 (27.3)	32 (25.8)	
Stage			
I	208 (56.1)	73 (58.9)	0.063
II	45 (12.1)	6 (4.8)	
III	82 (22.1)	27 (21.8)	
IV	36 (9.7)	18 (14.5)	
NA	2	0	
Metastasis			
M0	205 (97.2)	71 (95.9)	0.7
M1	6 (2.8)	3 (4.1)	
NA	162	50	
N classification			
N0	182 (54.8)	44 (38.3)	0.002
N1	150 (45.2)	71 (61.7)	
NA	41	9	
T classification			
T1	112 (30.1)	30 (24.4)	0.095
T2	129 (34.7)	36 (29.3)	
T3	118 (31.7)	48 (39.0)	
T4	13 (3.5)	9 (7.3)	
NA	1	1	
Pathologic type			
Classical	256 (68.6)	97 (78.2)	0.012
Follicular	88 (23.6)	14 (11.3)	
Tall cell	23 (6.2)	12 (9.7)	
Others	6 (1.2)	1 (0.2)	
BRAF			
Wild-type	150 (41.4)	46 (38.3)	0.592
Mutated	212 (58.6)	74 (61.7)	
NA	11	4	
RAS			
Wild-type	330 (91.2)	113 (94.2)	0.34
Mutated	32 (8.8)	7 (5.8)	
NA	11	4	

### Analysis of related genes and functions of NUSAP1

**Fig. 4:**
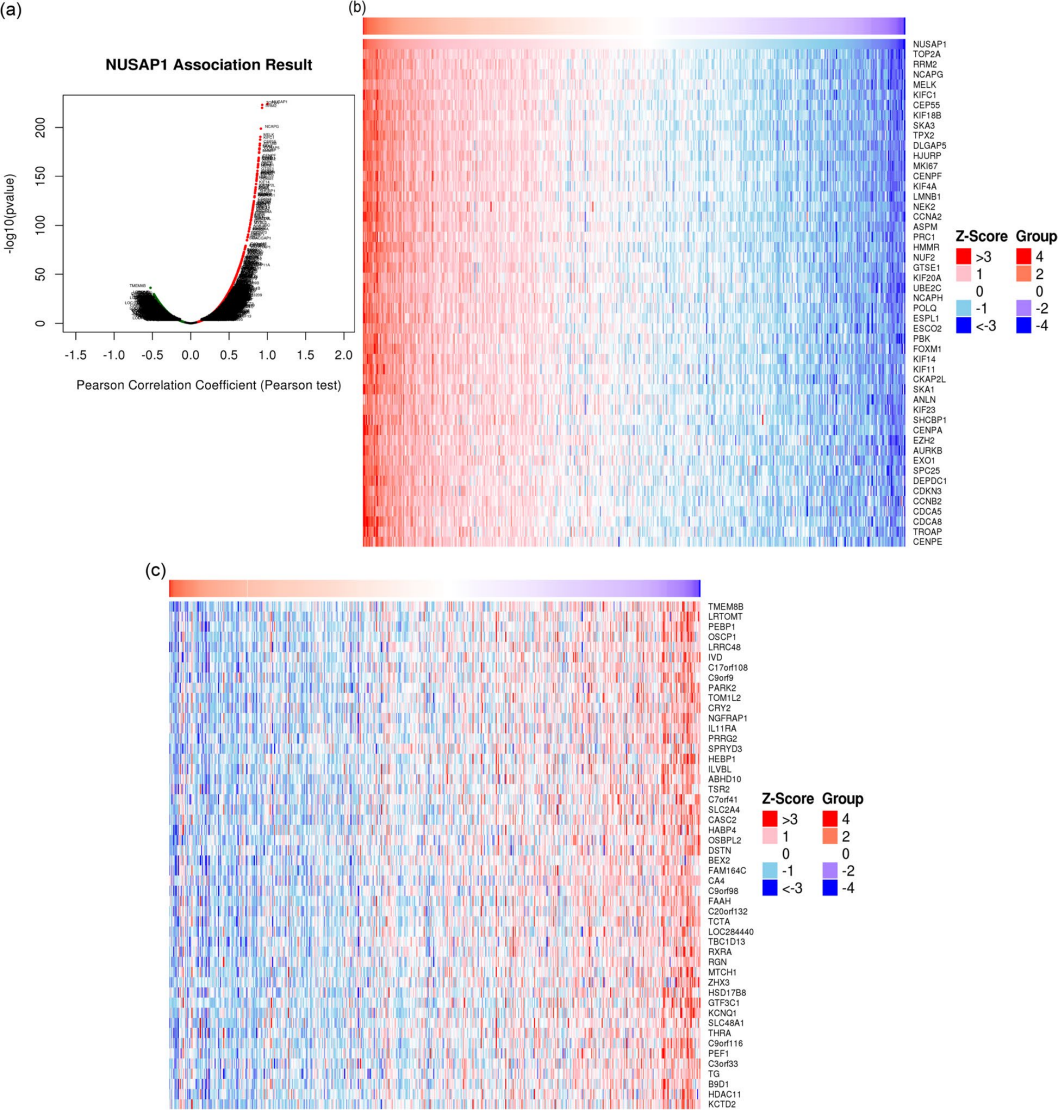
Related genes of NUSAP1. **a** A volcano map depicts the genes closely related to NUSAP1. A heat map shows the top 50 genes positively related to NUSAP1 (**b**) and the top 50 genes negatively related to NUSAP1 (**c**). Red and blue represent positively correlated genes and negatively correlated genes, respectively

**Fig. 5:**
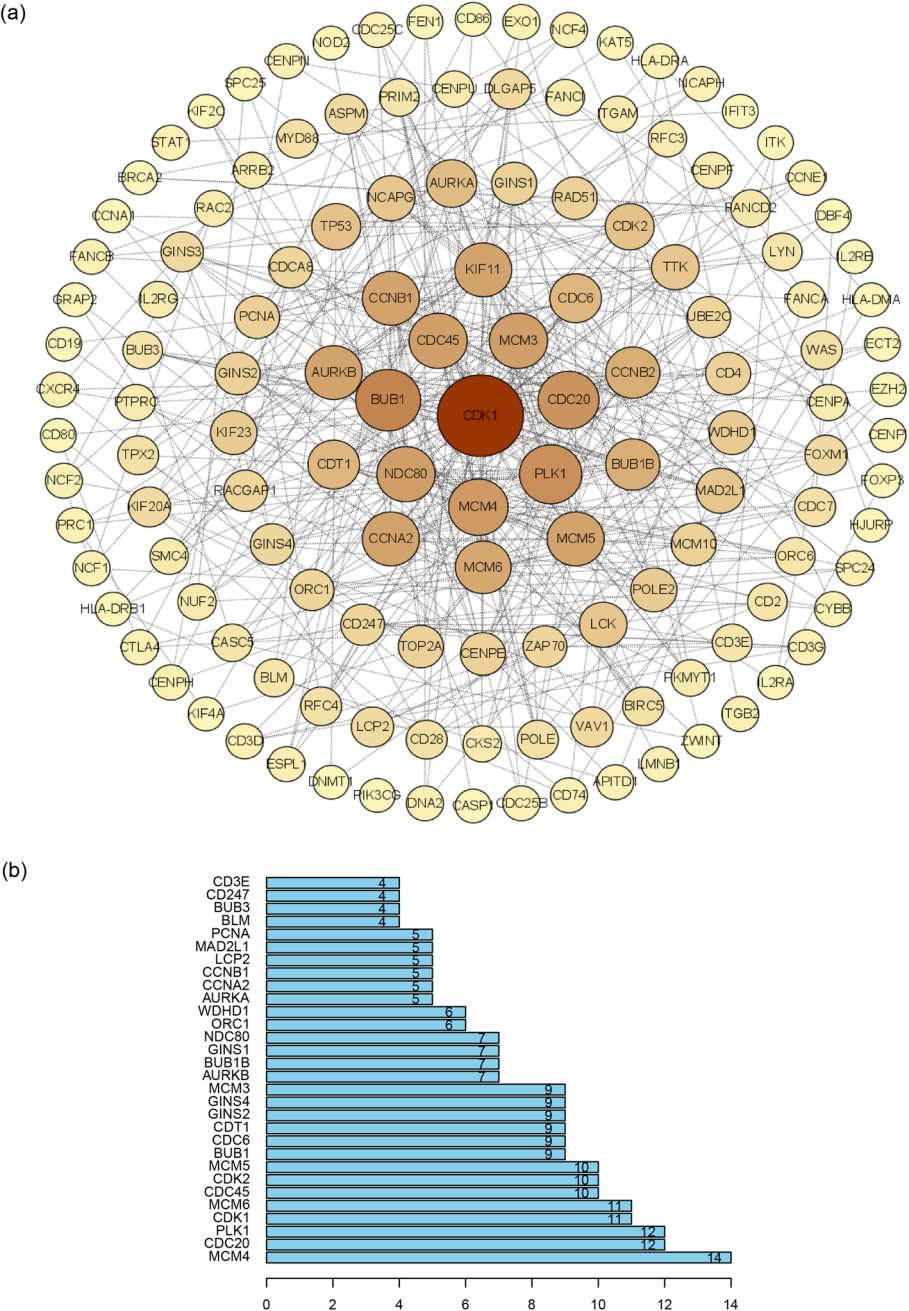
Identification of hub proteins from the PPI network. **a** PPI network of 2000 NUSAP1-related proteins and degree ≥ 2. The bigger the circle means the bigger the degree. **b** Histogram of the first 30 hub proteins

**Fig. 6:**
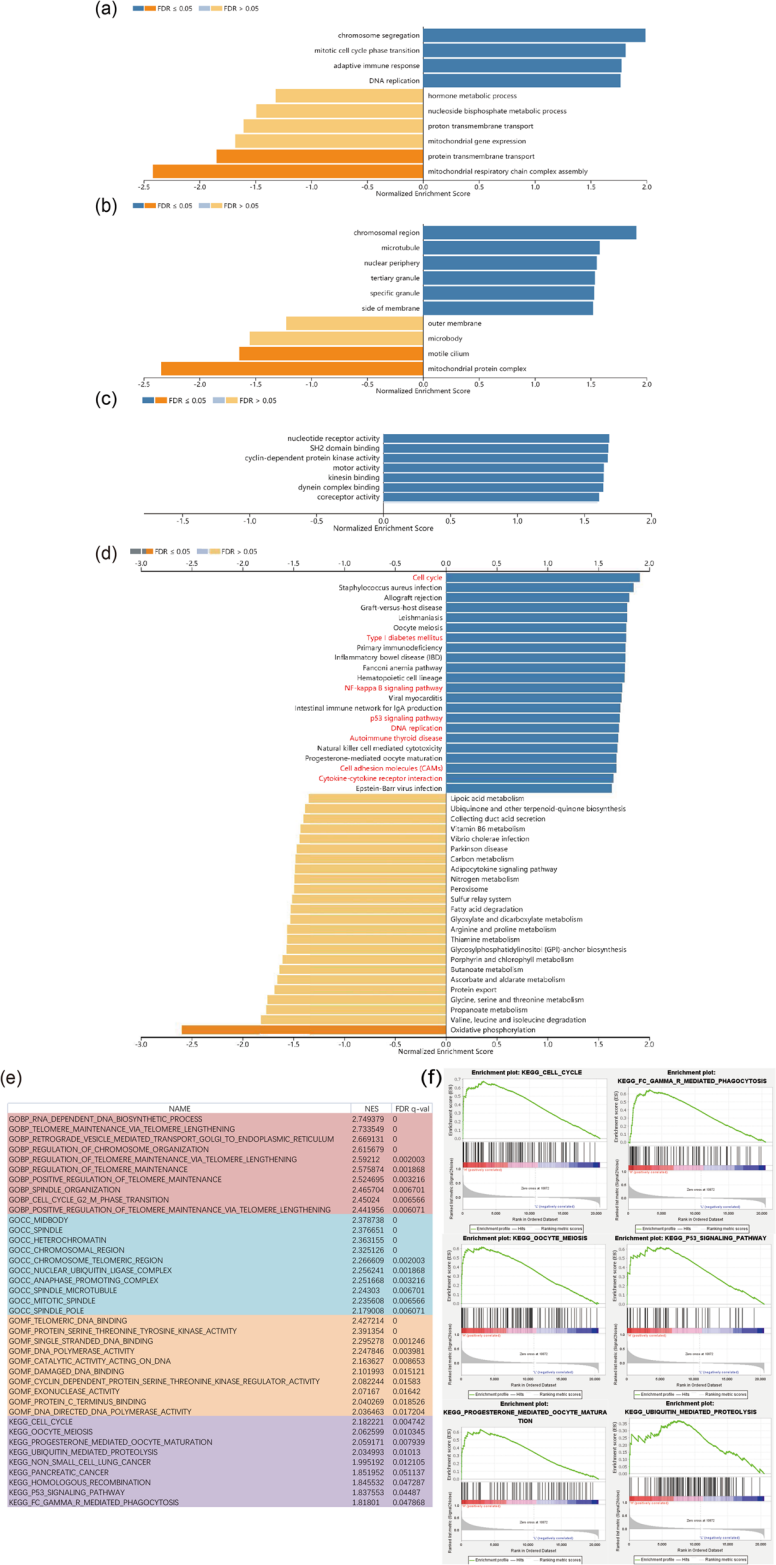
GO and KEGG analysis of NUSAP1. The histogram shows the GO (**a**–**c**) and KEGG (**d**) results of NUSAP1 in the PTC organization. **e** BP, CC, MF, and KEGG results of NUSAP1 in the H-NUSAP1 group. NES, normalized enrichment score. FDR *q*-val means multi-checked *p*-value. **f** A depiction of the main pathways in which NUSAP1 participates in the H-NUSAP1 group

Next, we aimed to study the function of NUSAP1 and related genes. A total of 501 cases of PTC in the THCA dataset were analyzed through the LinkedOmics website. The results of the study showed that a total of 4281 genes were negatively correlated with NUSAP1 expression, and 6195 genes were positively correlated (*p* < 0.01); these data are visualized as a volcano graph (Fig. 4a). Heat maps were used to visualize the top 50 positively correlated and top 50 negatively correlated genes that were most closely connected to NUSAP1 (Fig. 4b, c). In order to analyze the interaction with NUSAP1-related proteins, 2000 proteins closely linked to NUSAP1 were visualized through String and Cytoscape, and the first 30 core proteins were displayed using R language, including MCM4, CDC20, PLK1, CDK1, MCM6, CDC45, CDK2 MCM5, BUB1, CDC6, and (Fig. 5a, b). We next analyzed the role of NUSAP1 in PTC; GO and KEGG analysis were performed by GSEA. The results showed a positive correlation with biological processes (BP) (Fig. 6a) related to mitotic cell cycle phase transition and adaptive immune response; a negative correlation was found for BP related to protein transmembrane transport and mitochondrial respiratory chain complex assembly. For the cell component (CC) (Fig. 6b), the chromosomal region was positively correlated; negatively related BP included motility and mitochondrial protein complex. For molecular function (MF) (Fig. 6c), BP primarily associated with nucleic acid receptor activity and cyclin-dependent protein kinase activity were positively correlated. For KEGG (Fig. 6d), positive correlations were identified for cytokine-cytokine receptor interaction, cell cycle, type I diabetes mellitus, NF-kappa B signaling pathway, p53 signaling pathway, DNA replication, autoimmune thyroid diseases, and cell adhesion molecules (CAMs); a negative correlation was identified for oxidative phosphorylation. To learn more about the NUSAP1 function in the H-NUSAP1 group, GO and KEGG analysis were performed by GSEA (Fig. 6e). The most likely related KEGG pathways (Fig. 6f) included “CELL_CYCLE” and “P53_SIGNALING_PATHWAY.”

### The relationship between NUSAP1 and tumor microenvironment

**Fig. 7:**
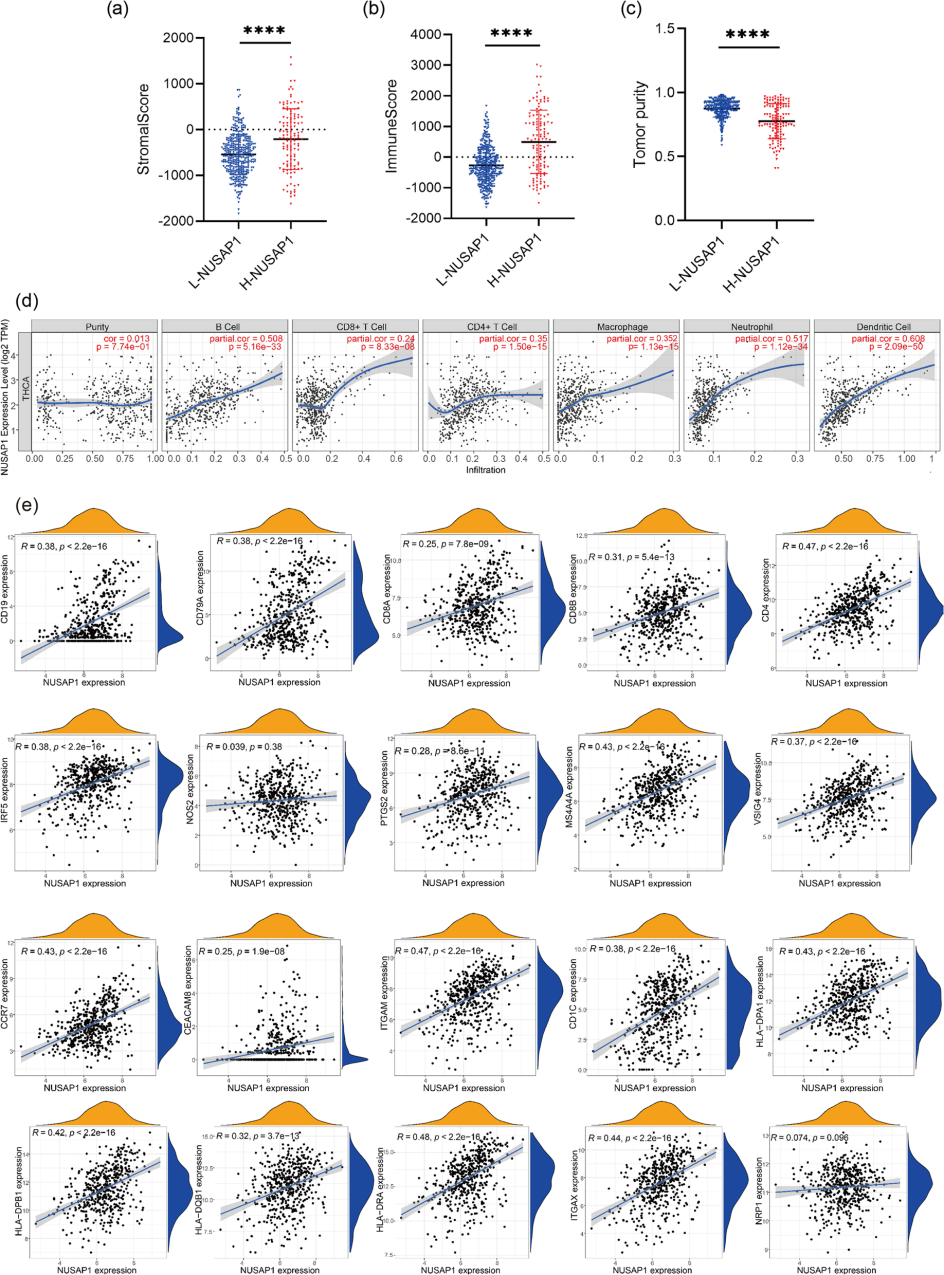
Prediction of the relationship between NUSAP1 and immune infiltration. ESTIMATE was used to analyze the stromalScore (**a**), immuneScore (**b**), and tumor purity (**c**) in the L-NUSAP1 and H-NUSAP1 groups. **d** TIMER was applied to examine the correlation between NUSAP1 and six immune cells in the THCA dataset. **e** A scatter plot was used to visualize the correlation between NUSAP1 and immune molecular markers in PTC. The yellow part represents the density map of the expression of NUSAP1, and the blue part represents the density map of the expression of each immune molecular marker. *p < 0.05, **p < 0.01, ***p < 0.001, ****p < 0.0001

To investigate the association between NUSAP1 and the tumor microenvironment, we first calculated stromalScore, immuneScore, and tumor purity in PTC tissues using the ESTIMATE method. The stromalScore and immuneScore were much higher in the H-NUSAP1 group than in the L-NUSAP1 group (both *p*-values were < 0.0001) (Fig. 7a, b). Moreover, the tumor purity level in the L-NUSAP1 group was substantially lower than that in the H-NUSAP1 group (Fig. 7c). We used a histogram to see the proportion of immune cells in PTC tissue (Additional file 1: Fig. S2). In order to analyze the relationship between NUSAP1 and immune cells, the correlation between NUSAP1 and B cells, dendritic cells, CD4+ T cells, CD8+ T cells, macrophages, and neutrophils in PTC was analyzed by TIMER. The correlation coefficients of NUSAP1 and the abovementioned immune cells, respectively, were 0.508, 0.608, 0.35, 0.24, 0.517, and 0.352 (*p* < 0.0001, Fig. 7d). Heat maps were used to show the interaction between different immune cells in PTC (Additional file 1: Fig. S3). To further analyze the relationship between NUSAP1 and immune cells, we first needed to better understand the changes in immune cell molecular markers. The results (Fig. 7e) revealed positive associations for all, including B cell molecular markers (CD19 and CD79A), CD8+ T cell molecular markers (CD8A, CD8B), CD4+ T cell molecular markers (CD4), M1 macrophage molecular markers (IRF5 and PTGS2), M2 macrophage molecular markers (CD163, VSIG4, and MS4A4A), neutrophil molecular markers (CEACAM8, ITGAM, and CCR7), and dendritic cell molecular markers (HLA-DPB1, HLA-DQB1, HLA-DRA, HLA-DPA1, CD1C, and ITGAX). NUSAP1 was not correlated with the M1 macrophage molecular marker NOS2 or the dendritic cell molecular marker NRP1.

### Construction of ceRNA network

**Fig. 8:**
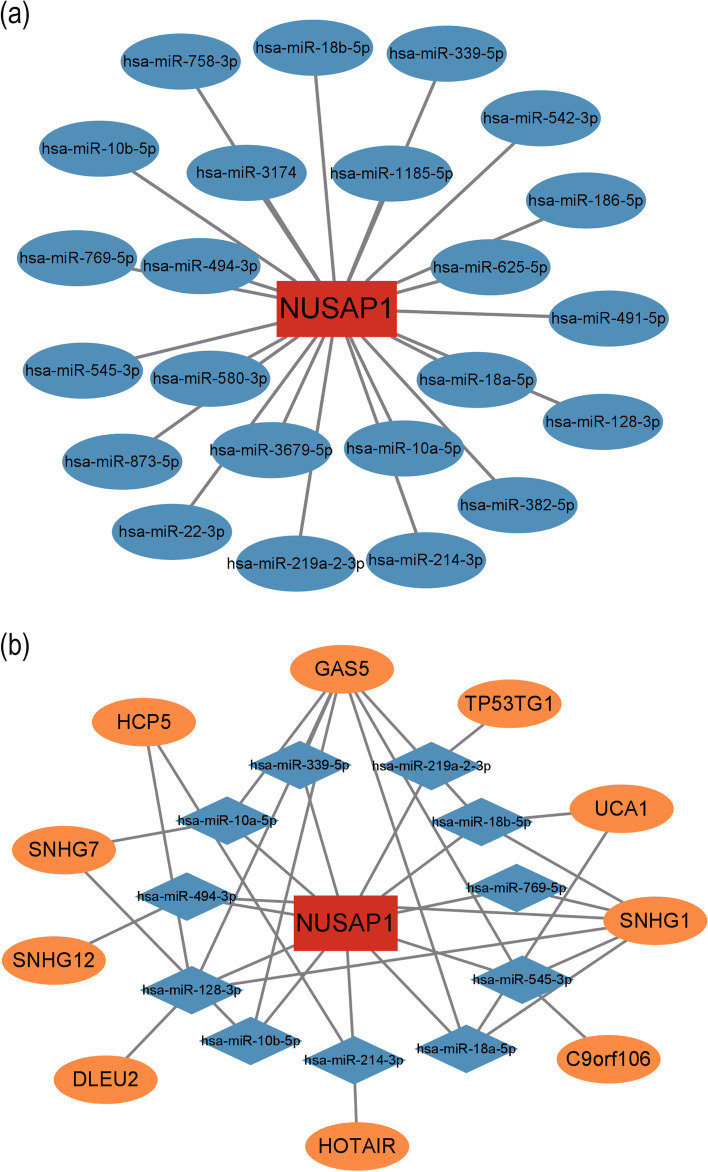
Identification of potential miRNAs and lncRNAs that bind to NUSAP1 in PTC. **a** Results of NUSAP1 targets predicted by StarBase V2.0. **b** The network of lncRNA–miRNA–mRNA. Orange indicates lncRNAs, blue indicates miRNAs, and red indicates NUSAP1

To better explore the different roles in NUSAP1-related miRNAs and lncRNAs of papillary thyroid carcinoma, we constructed mRNA–miRNA–lncRNA networks. By exploring the miRNAs that bind to NUSAP1 through the starbase website, we found that after removing duplicate values, 24 miRNAs had binding sites for NUSAP1 (Fig. 8a). Since miRNAs negatively regulate NUSAP1, we performed the miRNA expression quantification data in the TCGA to search for miRNAs that are underexpressed in PTC. Eleven miRNAs were identified. To further find related lncRNAs, we analyzed the lncRNAs bound to miRNAs again by starbase and ensured that lncRNAs were highly expressed in PTC then found GAS5, SNHG7, UCA1, SNHG1, HCP5, DLEU2, HOTAIR, TP53TG1, SNHG12, and C9orf106. Based on the above findings, we constructed a ceRNA network by cytoscape (Fig. 8b).

### The value of NUSAP1 in therapy

**Fig. 9:**
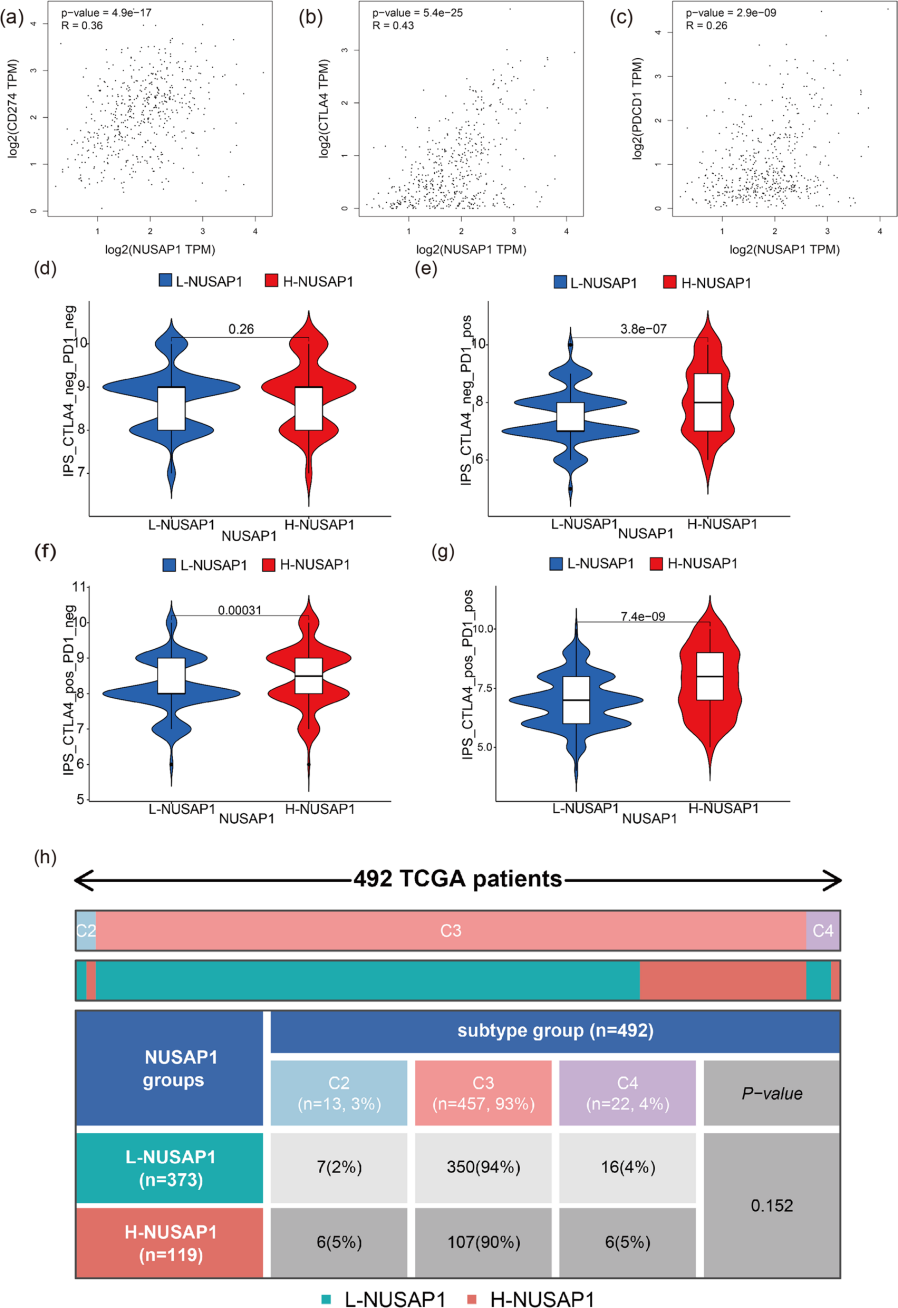
The relationship between NUSAP1 and immunotherapy. **a**–**c** Scatter plots illustrate the correlation between NUSAP1 and the CD274, CTLA4, and PDCD1 immune checkpoints in PTC. The violin chart shows the immunotherapy scores of the L-NUSAP1 and H-NUSAP1 groups without CTLA4 and PD1 treatment (**d**), PD1 treatment only (**e**), CTLA4 treatment only (**f**), and PD1 + CTLA4 treatment (**g**). White squares represent the first and third quartile ranges, black lines represent 95% confidence interval ranges, and widths represent probabilities. **h** A heat map and table include the immunotypes (C2, C3, and C4) of PTC in the L-NUSAP1 group and H-NUSAP1 group. Gray, pink, purple, green, and red represent C2, C3, C4, L-NUSAP1, and H-NUSAP1, respectively

The GEPIA website was utilized to evaluate the association between NUSAP1 and immune checkpoints (CD274, CTLA4, and PDCD1) in order to further study the effect of NUSAP1 on PTC immunotherapy. The results showed that the *R* value of NUSAP1 and CD274 was 0.36 (Fig. 9a, *p* < 0.0001), the *R* value of CTLA4 was 0.43 (Fig. 9b, *p* < 0.0001), and the *R* value of PDCD1 was 0.26 (Fig. 9c, *p* < 0.0001). On this premise, the treatment scores of the immune checkpoints were examined, and there were no statistical differences between the L-NUSAP1 group and the H-NUSAP1 group without CTLA4 and PD1 treatment (Fig. 9d). PD1 treatment alone (Fig. 9e) and CTLA4 treatment alone (Fig. 9f) showed higher immune scores in the H-NUSAP1 group than in the L-NUSAP1 group, and the difference was statistically significant (*p* < 0.0001). With combined PD1 and CTLA4 treatment (Fig. 9g), the immunotherapy score in the H-NUSAP1 group still had a higher immune score (*p* < 0.0001), but there were almost no statistically significant changes with respect to immune phenotyping (Fig. 9h).

To explore drugs related to NUSAP1, genes significantly associated with NUSAP1 were entered into the Cmap database to find the gene expression profiles of small molecules matching these genes. The top 10 related drugs/molecules are shown in Table 3 and sorted by score. The top negative enrichment scores belong to CDK inhibitors, including palbociclib and purvalanol-a.

**Table 3:** CMap analysis identified potential therapeutic drugs for the H-NUSAP1 group

Rank	Score	Type	Name	Description
1	− 99.93	cp	Palbociclib	CDK inhibitor
2	− 99.75	cp	Purvalanol-a	CDK inhibitor
3	− 99.65	cp	Aminopurvalanol-a	Tyrosine kinase inhibitor
4	− 99.61	cp	Floxuridine	DNA synthesis inhibitor
5	− 99.54	cp	L-690488	Inositol monophosphatase inhibitor
6	− 99.33	cp	Bisbenzimide	DNA-binding agent
7	− 99.19	cp	JAK3-inhibitor-VI	JAK inhibitor
8	− 99.08	cp	Pyrimethamine	Dihydrofolate reductase inhibitor
9	− 99.01	cp	Selumetinib	MEK inhibitor
10	− 99.01	cp	Teniposide	Topoisomerase inhibitor

## Discussion

The rapid development of sequencing technology has promoted our understanding of pan-cancer, especially for thyroid cancer [31, 32]. In this analysis, we first identified the differential expression of NUSAP1 in pan-cancer using TIMER and validated it in KMplot. High NUSAP1 expression is associated with poor disease prognosis, and NUSAP1 is interrelated to immune cell abundance. Using public databases, we found that the level of NUSAP1 expression in PTC tissues was significantly higher than in normal tissue, showed good diagnostic value, was closely related to the prognosis of PTC patients, and was an independent risk factor for PTC. Therefore, we completed an in-depth study of the potential functions of NUSAP1 and discovered that it was related to cell proliferation and immune response. The analysis of NUSAP1 and the tumor microenvironment lays the foundation, and the analysis results were consistent with expectations. In the H-NUSAP1group, stromal and immune cell infiltration is likely to be greater, and tumor purity is possible to be less. We also constructed a lncRNA–miRNA–mRNA network. Considering that NUSAP1 is closely relevant to immune infiltration, it is speculated that NUSAP1 is related to the efficacy of immunosuppressive agents. We used TCIA data to analyze that PD1 immunotherapy and CTLA4 immunotherapy may be better in the H-NUSAP1group and screened out several potential small molecule drugs that can reverse NUSAP1.

NUSAP1 is highly abundant in many cancers, is related to a short OS period, and has a significant positive correlation with six kinds of immune cells. Therefore, it is conjectured that NUSAP1 also performs certain biological functions in thyroid cancer. Li et al. [33] found that the expression of NUSAP1 was notably increased in anaplastic thyroid carcinoma (ATC). Therefore, we used the THCA dataset to analyze the expression level of NUSAP1 in PTC. The results showed that NUSAP1 was also increased significantly in PTC tissues and in four GEO databases. The same results are seen in all datasets. In recent years, studies have reported that NUSAP1 was involved in malignant pleural mesothelioma (MPM) [34], liver cancer [35], prostate cancer [36], and other cancers, with diagnostic value. We used the THCA dataset and analyzed the GSE27155, GSE33630, GSE58545, and GSE60542 datasets to demonstrate that NUSAP1 has a better diagnostic value in PTC. In PTC, patients with high NUSAP1 levels have shorter PFS and are prone to relapse. In NSCLC [37], LIHC [38], and other cancers, NUSAP1 is associated with a poor prognosis of the disease. We demonstrated that in PTC, NUSAP1 is an independent risk factor. It was reported that central lymph node metastasis (CLNM) was a risk factor for poor prognosis and recurrence of papillary thyroid microcarcinoma [39]. In addition, patients with high NUSAP1 expression are more likely to develop lymph node metastasis, and these patients primarily have typical pathological types of PTC, which further illustrates the potential of NUSAP1 to serve as a prognostic marker.

We found that NUSAP1 interacts with cell cycle-related proteins, such as MCM4, CDC20, and PLK1. In addition, we used GSEA analysis to determine that NUSAP1 may affect the cell cycle, DNA replication, cell adhesion, the NF-KB signaling pathway, and autoimmune thyroid disease (AITD); high expression of NUSAP1 does indeed participate in the cell cycle pathway. In addition, it was also discovered that high expression of NUSAP1 is linked to ubiquitination-mediated protein degradation. A recent protein genomics study on PTC reported that the E3 ubiquitin ligase HUWE1 and DUB were upregulated in tumors and metastatic disease [40]. According to reports, silencing NUSAP1 inhibited proliferation, migration, and invasion of bladder cancer cells [41]. Zhao et al. [9] found that NUSAP1 was involved in GBM cell proliferation, apoptosis, and DNA damage in patients with malignant glioma (GBM) and was related to GBM chemotherapy resistance. Autoimmunity and inflammation are risk factors for PTC. Zhou et al. and Li et al. [42, 43] reported that HT was a protective factor for PTC, and high TPOAb concentrations were less prone to CLNM. However, it has also been suggested that high urinary iodine, as a risk factor for HT, promotes CLNM of PTC [44]. At present, the exact mechanism of the association between AITD and PTC remains unclear [45]. We observed that NUSAP1 participates in AITD, which provides a theoretical basis for exploring the relationship between AITD and PTC.

As we all know, immune cells that infiltrate a tumor affect the tumor microenvironment. The tumor microenvironment has a complex impact on the cancer cells with respect to invasion, proliferation, and migration. In liver cancer, Chen et al. and Huang et al. [38, 46] reported that NUSAP1 expression was closely related to immune cells. Our study also found that NUSAP1 was positively correlated with dendritic cells, B cells, macrophages, CD8+ T cells, CD4+ T cells, and neutrophils. However, high expression of NUSAP1 is related to the poor prognosis of PTC, and it is speculated that NUSAP1 may be related to immune escape. This indicates that NUSAP1 plays a complex change in tumor immunity.

mRNAs act as a sponge for miRNAs and are regulated by miRNAs [47]. Starbase is the most common tool for predicting mRNA-binding miRNAs. We revealed a total of 24 miRNAs with binding sites to NUSAP1, and hsa-miR-10a-5p, hsa-miR-10b-5p, hsa-miR-18a-5p, hsa-miR-18b-5p, hsa-miR-128-3p, hsa-miR-214-3p, hsa-miR-219a-2-3p, hsa-miR-339-5p, hsa-miR-494-3p, hsa-miR-545-3p, and hsa-miR-769-5p were lowly expressed in PTC. Chen et al. [10] verified the binding site of hsa-miR-769-5p and NUSAP1 by luciferase gene reporter gene detection, and NUSAP1 can reverse the cell growth, migration, invasion, and apoptosis caused by overexpression of hsa-miR-769-5p in bladder cancer. NUSAP1 is also regulated by hsa-miR-18b-5p and affects the proliferation of hepatocellular carcinoma [48]. We constructed a ceRNA network by finding lncRNAs that bind to miRNAs and identified a total of 10 lncRNAs. It is found that lncRNAs can bind to multiple miRNAs, among which GAS5 binds the most miRNAs. GAS5, SNHG7, UCA1, SNHG1, HCP5, DLEU2, HOTAIR, and SNHG12 were reported to be differentially expressed in PTC and involved in malignant behaviors [49–56].

Next, we analyzed the interaction between NUSAP1 and three common immune checkpoint molecules and revealed that NUSAP1 was mainly related to CD274 and CTLA4, with a low correlation to PDCD1. However, the expression level of NUSAP1 is related to PD1 immunotherapy scores and CTLA4 immunotherapy scores. The treatment scores were all positively correlated, and it was also positively correlated with PD1 and CTLA4 combined treatment. Anti-cancer immunotherapy [57], especially immune checkpoint inhibitors, can release the immune system and activate cytotoxic lymphocytes to kill cancer cells. Consistent with the research of Thorsson et al. [58], our research also observed that PTC was mainly C3 (inflammatory type), but we found that high expression of NUSAP1 was not different from the immunophenotyping of PTC. Based on the above results, we further predicted small-molecule drugs that might reverse the effect of NUSAP1, and finally discovered 10 small-molecule drugs. Similar to the above results, CDK inhibitors played an important role in reversing the effects of NUSAP1. In this class of drugs, two small-molecule drugs have been screened, including palbociclib and purvalanol-a. The antitumor effect of palbociclib had been widely reported. In addition, selumetinib increased radioiodine affinity in radioactive iodine (RAI)-refractory tumors, which exerted its effectiveness in advanced thyroid cancer [59].

Our analysis shows that NUSAP1 can be used as a diagnostic marker of PTC and can affect prognosis. Overexpression of NUSAP1 is associated with lymph node metastasis and recurrence of PTC. NUSAP1 may have an effect on tumor cell growth by affecting the cell cycle and DNA damage repair. Altering cell adhesion impacts the migration and invasion capabilities of tumor cells. This impacts PTC through altering AITD and has a wide range of prognostic values in pan-cancer. We created a NUSAP1-specific ceRNA network and predicted NUSAP1-related small-molecule drugs. However, the role of NUSAP1 in the tumor microenvironment is complex. It is positively correlated with a variety of immune cells and is also positively correlated with immune checkpoint molecules. It is speculated that NUSAP1 is related to immune escape. Therefore, further studies are required to determine the role of NUSAP1 in PTC to provide a basis for the user of NUSAP1 as a therapeutic target of PTC.

## Conclusions

Through a multifaceted analysis of the value and function of NUSAP1 in PTC, our study predicts that NUSAP1 may become a potential diagnostic and prognostic marker of PTC, as well as a predictive molecule for the efficacy of therapy. NUSAP1 is related to the development and recurrence of PTC and also provides a link between AITD and PTC. At the same time, NUSAP1 may be involved in immune escape and affect the tumor microenvironment, so immune checkpoint inhibitor therapy may have a better effect. However, further experiments are required to verify the above results.

## Supplementary Information

**Additional file 1: Figure S1.** Kaplan-Meier survival curves comparing the high and low expression of NUSAP1 in 5 types of cancer. Survival curves of OS in pancreatic ductal adenocarcinoma (PDAC), Kidney Renal Clear Cell Carcinoma (KIRC), Kidney renal papillary cell carcinoma (KIRP), Liver hepatocellular carcinoma (LIHC), Lung adenocarcinoma (LUAD). **Figure S2.** The proportion of immune cells in the PTC tissue of the THCA data set. The x-axis shows the sample name, and the y-axis shows the proportion of immune cells. **Figure S3.** Correlation between immune cells in PTC tissue of THCA data set. The content in the lower left corner displays the correlation coefficient, and the upper right corner visualizes the correlation coefficient. Blue represents positive correlation, red represents negative correlation, and the darker the color, the higher the correlation.

## Data Availability

The data used to support this study are available publicly in the University of California at Santa Cruz (UCSC) database and Gene Expression Omnibus (GEO).

## Abbreviations

NUSAP1: Nucleolar spindle-associated protein 1
PTC: Papillary thyroid carcinoma
OS: Overall survival
PFS: Progression-free survival
FVPTC: Follicular papillary thyroid carcinoma
AIT: Autoimmune thyroiditis
YAP1: Yes1-associated transcriptional regulator
GSK-3β: Glycogen synthase kinase 3 beta
ICB: Immune checkpoint blockade
TCGA THCA: The Cancer Genome Atlas Thyroid Cancer
VarScan: Variation data
UCSC: University of California at Santa Cruz
NCBI: National Center for Biotechnology Information
GEO: Gene Expression Omnibus
TIMER: Tumor Immune Estimation Resource
GEPIA: Gene Expression Profiling Interactive Analysis
GTEx: Genotype-Tissue Expression Project
ROC: Receiver operating characteristic
K-M: Kaplan-Meier
CPTAC: Clinical Proteomics Tumor Analysis Consortium
GSEA: Gene set enrichment analysis
PRAD: Prostate adenocarcinoma
KICH: Kidney chromophobe
KIRP: Kidney renal papillary cell carcinoma
LIHC: Liver hepatocellular carcinoma
LUAD: Lung adenocarcinoma
TMB: Tumor mutation burden
BP: Biological processes
CC: Cell component
MF: Molecular function
CAMs: Cell adhesion molecules
ATC: Anaplastic thyroid carcinoma
MPM: Malignant pleural mesothelioma
AITD: Autoimmune thyroid disease

## Footnotes

1 https://xena.ucsc.edu/

2 http://www.ncbi.nlm.nih.gov/geo/

3 http://gepia.cancer-pku.cn/

4 http://www.linkedomics.org/

5 https://string-db.org/

6 https://cistrome.shinyapps.io/timer/

7 https://tcia.at/
