# Supplementary material for: Evaluate the diagnostic and prognostic value of NUSAP1 in papillary thyroid carcinoma and identify the relationship with genes, proteins, and immune factors
Source: World J Surg Oncol. 2022 Jun 16;20:207. doi: 10.1186/s12957-022-02652-9 (PMC9202173; doi:10.1186/s12957-022-02652-9)
Supplement: Supplementary file 1 — Additional file 1: Figure S1. Kaplan-Meier survival curves comparing the high and low expression of NUSAP1 in 5 types of cancer. Survival curves of OS in pancreatic ductal adenocarcinoma (PDAC), Kidney Renal Clear Cell Carcinoma (KIRC), Kidney renal papillary cell carcinoma (KIRP), Liver hepatocellular carcinoma (LIHC), Lung adenocarcinoma (LUAD). Figure S2. The proportion of immune cells in the PTC tissue of the THCA data set. The x-axis shows the sample name, and the y-axis shows the proportion of immune cells. Figure S3. Correlation between immune cells in PTC tissue of THCA data set. The content in the lower left corner displays the correlation coefficient, and the upper right corner visualizes the correlation coefficient. Blue represents positive correlation, red represents negative correlation, and the darker the color, the higher the correlation. [file 12957_2022_2652_MOESM1_ESM.docx]

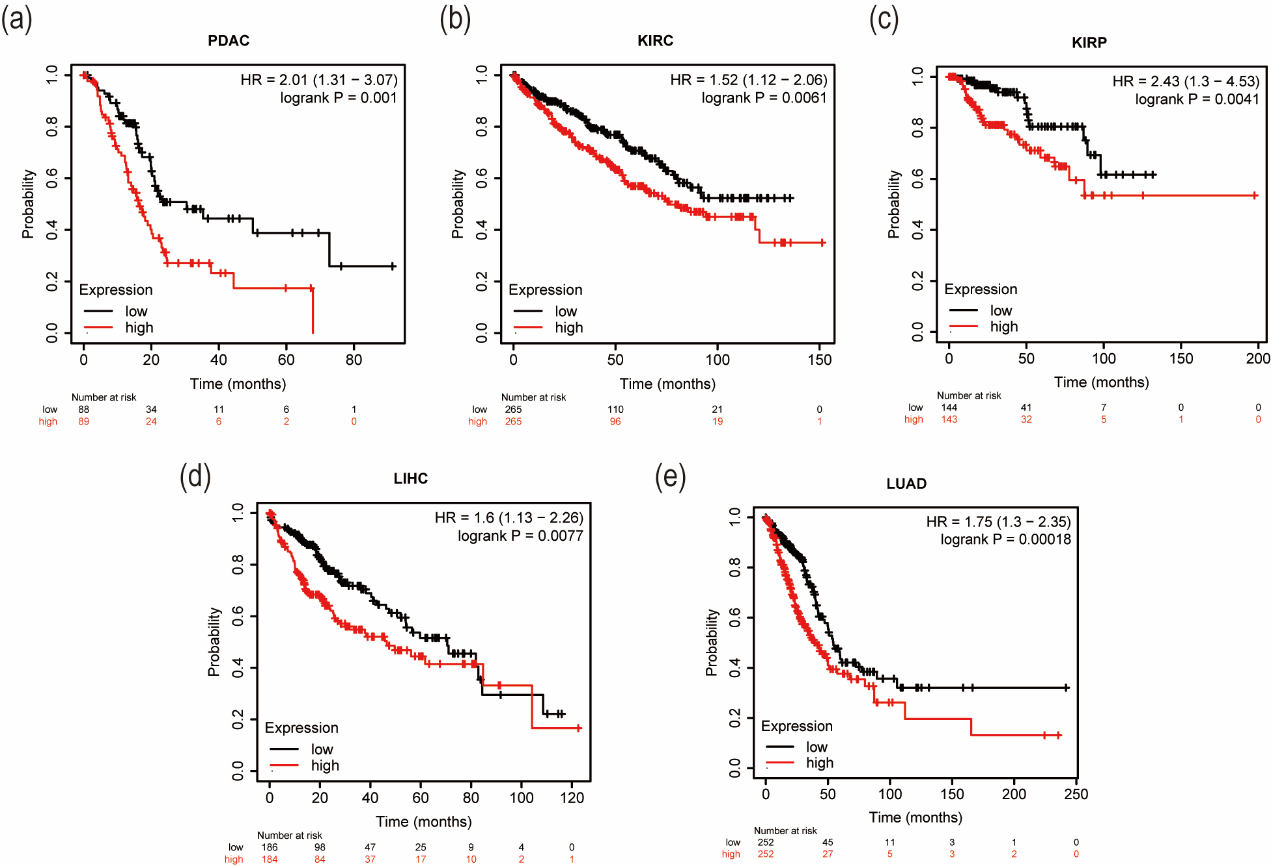


Figure S1: Kaplan-Meier survival curves comparing the high and low expression of NUSAP1 in 5 types of cancer. Survival curves of OS in pancreatic ductal adenocarcinoma (PDAC), Kidney Renal Clear Cell Carcinoma (KIRC), Kidney renal papillary cell carcinoma (KIRP), Liver hepatocellular carcinoma (LIHC), Lung adenocarcinoma (LUAD).


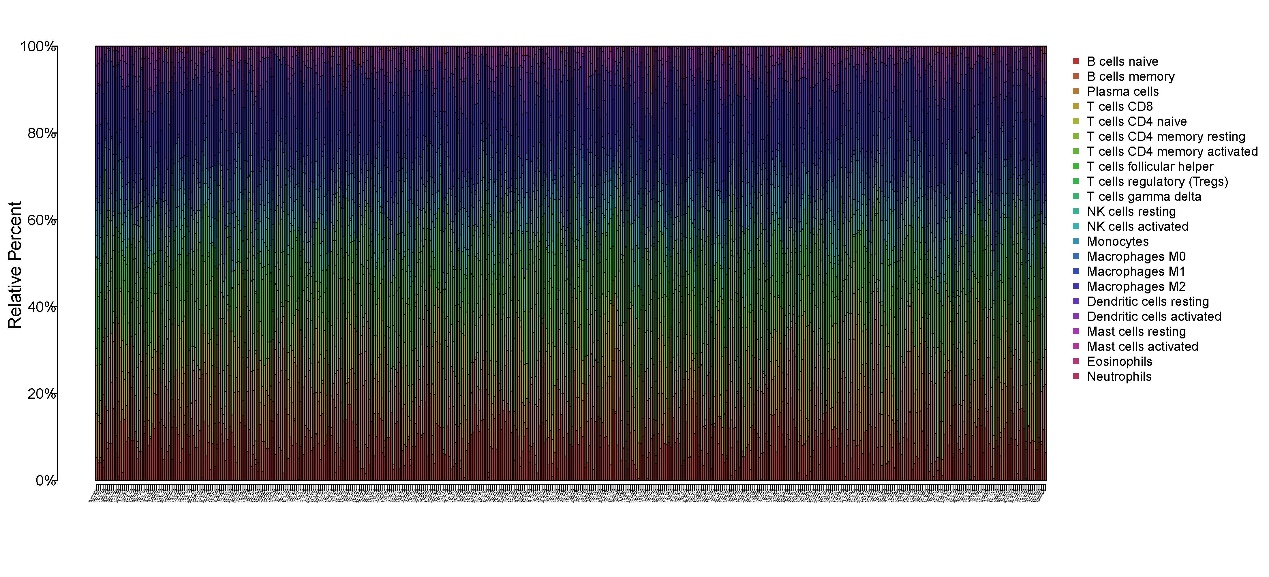


Figure S2: The proportion of immune cells in the PTC tissue of the THCA data set. The x-axis shows the sample name, and the y-axis shows the proportion of immune cells.


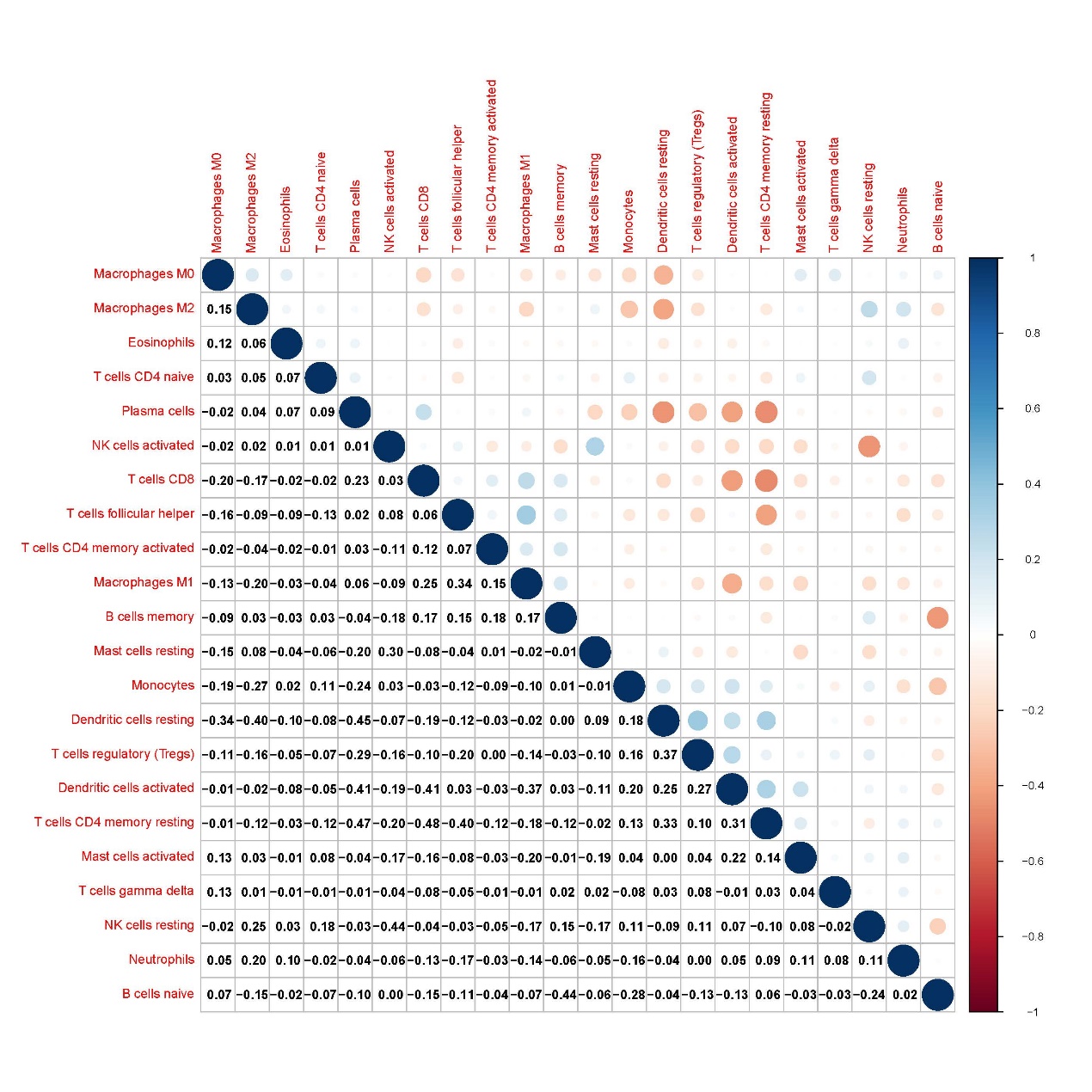


Figure S2: Correlation between immune cells in PTC tissue of THCA data set. The content in the lower left corner displays the correlation coefficient, and the upper right corner visualizes the correlation coefficient. Blue represents positive correlation, red represents negative correlation, and the darker the color, the higher the correlation.
